# The effect of high-fat diet and 13-cis retinoic acid application on lipid profile, glycemic response and oxidative stress in female Lewis rats

**DOI:** 10.1371/journal.pone.0238600

**Published:** 2020-09-18

**Authors:** Ivana Ilić, Nada Oršolić, Edi Rođak, Dyana Odeh, Marko Lovrić, Robert Mujkić, Marija Delaš Aždajić, Anđela Grgić, Maja Tolušić Levak, Martin Vargek, Branko Dmitrović, Tatjana Belovari

**Affiliations:** 1 Department of Anatomy, Histology, Embryology, Pathological Anatomy and Pathological Histology, Faculty of Dental Medicine and Health, Josip Juraj Strossmayer University of Osijek, Osijek, Croatia; 2 Department of Histology and Embryology, Faculty of Medicine Osijek, Josip Juraj Strossmayer University of Osijek, Osijek, Croatia; 3 Department of Animal Physiology, Faculty of Science Zagreb, University of Zagreb, Zagreb, Croatia; 4 Department of Dermatology and Venereology, Sestre Milosrdnice University Hospital Center, Zagreb, Croatia; 5 Department of Dermatology and Venereology, University Hospital Osijek, Osijek, Croatia; 6 Division of Gastroenterology and Hepatology, Department of Internal Medicine, Medical University of Graz, Graz, Austria; 7 Clinical Institute of Pathology and Forensic Medicine, University Hospital Osijek, Osijek, Croatia; Max Delbruck Centrum fur Molekulare Medizin Berlin Buch, GERMANY

## Abstract

Vitamin A and its metabolites are key regulators of the development of adipose tissue and associated metabolic complications. The aim of this study was to determine the effect of high fat diet and 13-cis retinoic acid (13 cRA) application on metabolic parameters, adipogenic and inflammatory indicators in female Lewis rats. Female rats of Lewis strain were fed standard laboratory diet (STD) and high fat diet (HFD, 45% of saturated fatty acids) during 30 days. The groups were divided into additional 3 groups (6 rats each): two experimental groups that received 13 cRA orally on a daily basis during 30 days (7.5 mg/kg and 15 mg/kg, respectively) and the control group that was given sunflower oil. Animals were sacrificed after 60 days. Feeding of Lewis rats with chronic HFD diet with 13 cRA supplementation increased weight gain, adiposity index, dyslipidaemia, hyperleptinaemia, insulin resistance, VLDL concentrations, oxidative stress and atherogenic indices. Administration of 13 cRA in Lewis rats fed STD did not change the weight of the animals, but it slightly increased the atherogenic parameters. 13 cRA and HFD affect metabolic parameters, glucose and lipid metabolism in Lewis rats and its administration has a completely different effect on metabolism in rats fed STD, highlighting the complex role of vitamin A supplementation in obesity. Other factors, such as genetics, age, sex, adipose tissue distribution, also must be taken into consideration.

## Introduction

Obesity is a chronic disease of multifactorial aetiology [1] and one of the most common modern diseases that affects an ever bigger part of world population. During the last few decades, the number of obese people reached pandemic proportions [2], with approximately one fifth of the adult U.S. population having metabolic syndrome (MetS). It has been postulated that variations in the development and consequences of obesity depend on genetic predisposition combined with various environmental factors that lead to a chronically unbalanced energy intake relative to its expenditure. “Obesogenic environment,” which includes easy, 24-hour access to high-energy food, and large portion sizes, as well as a social environment that promotes a sedentary lifestyle, is contributing to obesity [3].

Consumption of high fat diet (HFD) can provoke disorders associated with lipid metabolism, such as increased visceral fat, hyperlipidaemia, atherosclerosis and insulin resistance [4]. Besides HFD, many studies have shown that retinoids participate in glucose and lipid metabolism as well as adipogenesis by activating the nuclear retinoic acid receptor (RAR) and the retinoic X receptor (RXR). Endogenous retinoids are stored in the form of all-*trans* retinyl esters in the liver and other tissues [5]. Thus, the accumulation of body fat is not only related to energy intake, but also to the kind of consumed nutrients [6]. With their regulatory roles, dietary nutrients provide essential vitamins and other factors. However, the effects of individual micronutrients on the development of metabolic diseases are not fully understood.

Vitamin A has the ability of regulating key cellular processes: cell differentiation, cell cycle control, cell growth and differentiation by enzymes regulating the conversion of the alcohol form of vitamin A (retinol) in the first step to an aldehyde (retinaldehyde) and then to a carboxylic acid form (retinoic acid, RA). The first step (oxidation of retinol to retinaldehyde) is catalysed by different cytosolic alcohol dehydrogenases (ADHs) and retinol dehydrogenases (RDHs). The oxidation of retinaldehyde to RA is catalysed by retinaldehyde dehydrogenases (RALDH1, RALDH2, RALDH3) [7]. RA is not produced by all cells in the body at the same time, it occurs locally in or near the cells where it will ultimately be used. Also, it is under unique spatiotemporal pattern and varies among different tissues [7, 8]. Retinol is secreted from the liver, bound to retinol binding protein (RBP4) and made available to all cells, such as gut, retina, liver etc., as well as to the embryonic cells during development, in order to be converted in RA [7]. The two RA isomers are detected *in vivo*: the most abundant all-*trans*-RA and 13 cis RA (13 cRA), which is found in lower concentrations in mice as well as in humans [9] although some studies indicate that RA cannot be synthesized in animals and must be taken from their diet [10]. The conversion of retinal into RA is irrversible and considered to be the rate-limiting step in RA biosynthesis [11]. The physiological functions of retinol are mainly mediated by its active acid metabolite, RA, which regulates more than 650 gene expressions by activating nuclear receptors RAR and RXR. All-*trans* retinoic acid is the major bioactive component among the retinoids [12]. Another RA isomer, 9-*cis* RA, has not been isolated *in vivo*. All-*trans* RA bind to RARs, whereas 9-*cis* RA binds to both RARs and RXRs. In contrast, 13 cRA does not exhibit specific binding to RXRs, and has a 100-fold lower affinity to RARs than all-*trans* RA or 9-*cis* RA [11].

All-*trans* RA are responsible for most of well-established roles in physiological processes: embryonic development (development of hindbrain, spinal cord, heart, eye, skeleton, lung, pancreas, genitourinary tract), haematopoiesis, neurogenesis, cardiogenesis, reproduction, immune function, eye development and vision, and is one of key regulators of adipose tissue biology [7, 13, 14]. On the other hand, 13 cRA has a specific pharmacokinetic properties, such as longer half-life and higher peak plasma concentrations in comparison to other RA isomers in the body. 13 cRA is considered a storage form of biologically active all-*trans* RA or 9-*cis* RA and it is normally circulating retinoid in human blood making up 25% of RA isomers [15]. Despite the fact that 13 cRA can be synthesized *in vivo* and that modulates brain neurochemical systems, it has well established pharmacological application for many clinical indications.

13 cRA, also named Isotretinoin, is commonly used as chemoterapeutic tool, neuroblastoma treatment, squamous cell carcinoma and many other tumors, oral leukoplakia, cheilitis, xeroderma pigmentosum, pityriasis rubra pilaris, Darier's disease and keratosis palmaris, rosacea, and seborrheic dermatitis [16, 17]. Furthermore, increased 13 cRA levels are associated with neurological side effects, such as depression, anxiety and irritability as well as suicidal attempts [15]. Overall, the most common and best known clinical indication for 13 cRA use is the treatment of recalcitrant nodular acne in adolescents [17].

Some data showed that vitamin A and its metabolites (retinyl esters, retinol, retinal, retinoic acid, oxidized and conjugated metabolites of both retinol and retinoic acid) regulate visceral fat loss of obese strains through activation of thermogenic and glucocorticoid pathways [18], thus ameliorating obesity and its related disorders, such as insulin resistance. RA potently blocks adipogenesis of cultured preadipose cells when introduced at early stages of the differentiation process. On the other hand, other reports indicate that all *trans*-RA at low doses may promote adipogenesis [19]. According to Granados et al., vitamin A intake during the early stages of postnatal life favours subsequent HFD-induced adiposity gain through mechanisms that may relate to changes in adipose tissue development, likely mediated by RA [20].

Excess adipose tissue accumulation is a precursor of pro-inflammatory cytokine production which contributes to obesity-related complications such as type 2 diabetes, glucose intolerance, insulin resistance, impaired blood pressure and hypertension, cardiovascular diseases, atherosclerosis, dyslipidaemia, chronic kidney disease, proinflammatory status and chronic inflammation in the body [21]. Zarei et al. confirmed consequent dyslipidaemia following RA administration in an animal model of Wistar rats [22]. Furthermore, irregular production of adipokines in obesity induces the production of reactive oxygen species (ROS), inciting the process known as oxidative stress, thus aggravating metabolic alterations [1].

Due to the fact that Isotretinoin is widley used in acne therapy among young people, it is worth investigating the long-term effect of 13 cRA in combination with different diets on weight changes, fat distribution, glucose metabolism, haematological and biochemical parameters, hormone disbalance, oxidative stress markers and atherogenic indices. According to Sengupta [23], one human year almost equals two rat weeks (13.8 rat days), so we used rats whose age corresponds to the early pubertal stage of humans since young people are prone to eating high-calorie diets and are exposed to the “obesogenic environment” on a daily basis.

## Materials and methods

### Animal model

Female Lewis rats, weighing 200-250g (n = 36), approximately 4–6 months old, obtained from the Department of Animal Physiology, Faculty of Science, University of Zagreb, were used in this study. A maximum of 4 animals was kept in a single cage and they were maintained on STD (Standard Diet 4RF21 GLP certificate, Mucedola, Italy) or HFD (45% energy from fat, GLP certificate, Mucedola, Italy) with water *ad libitum*. The animals were kept under 12L:12D h light-dark regime at 60% humidity. The ethical committee (Faculty of Science, University of Zagreb, Croatia) approved the present study (approval code ref. no.: 251-58-10617-19-284) for all of the animal protocols.

Animal studies were performed in compliance with the guidelines in force in the Republic of Croatia (the Croatian Animal Protection Law (Official Gazette “Narodne Novine”, 135/2006 and 37/2013), the Directive of The European Parliament and of the Council (2010/63/EU), as well as the Guide for the Care and Use of Laboratory Animals, DHHS Publ. (NIH) 86–123, 1985.

Animal experiments were carried out in accordance with EU Directive 2010/63 on the protection of animals used for scientific purposes.

### Study design

Animals (n = 36) were divided into two groups: standard laboratory chow diet (STD, Complete food for mice and rats 4RF21 Repelletted, Mucedola, Settimo Milanese, Italy) or high fat diet (HFD, Complete food for rodents purified diet 45% energy from fats, Mucedola, Settimo Milanese, Italy), 18 animals per group. The STD composition was crude protein (18.5%), crude oils and fats (3%), crude fibres (6%), crude ash (7%), whereas the high fat diet was comprised of differently proportioned percentages: crude protein (21%), crude oils and fats (23%), crude fibres (4.5%), crude ash (3.5%). Details of components and supplements (vitamins and minerals) of both diets (STD and HFD) are shown in Table 1.

**Table 1:** Composition of components and supplements of STD and HFD.

Analytical Components and Supplements (Vitamins and Minerals)		
	STD	HFD
E672 (vitamin A)	14400 I.U.	7600 I.U.
E671 (vitamin D3)	1260 I.U.	1900 I.U.
E1 (Fe)	180 mg	49.5 mg
E5 (Mn)	54 mg	13 mg
E6 (Zn)	67.5 mg	41.2 mg
E4 (Cu)	11.7 mg	7.4 mg
E2 (I)	0.9 mg	0.26 mg
3d302 (Co)	0.63 mg	0.19 mg
crude proteins	18.5%	0.19%
crude fats and oils	3%	21%
crude fiber	6%	23%
crude ash	7%	4.5%

Female Lewis (36) rats were used in the experiment and divided into two groups. The animals were individually labelled and weighed both before the beginning, as well as during the experiment. They were grouped based on similar body weight (± 10 g). The amount of 13 cRA was determined for each group during the experiment based on the animal mass.

The animals were fed according to their respective diets for 60 days (9 weeks), based on previously published literature where it was noted that a six-week high-fat diet causes metabolic alterations in male rats [24]. The food and water were assessed *ad libitum*.

Pathophysiological changes were induced by oral administration of RA in two different concentrations: 7.5 mg/kg and 15 mg/kg for 30 days by mixing the content of Roaccutane® capsules (Roche, France) with sunflower oil. Rats received daily gavages each morning between 10:00 and 12:00 am for 30 consecutive days. The control group was given sunflower oil during a 30-day period.

13 cRA was administered on a daily basis during 30 days by a single intragastric application. These two concentrations of 13 cRA were selected in accordance with the published literature which shows that a four-week administration of 13 cRA in rats leads to the first signs of body weight changes, changes in the oestrous cycle in female rats, changes in weight ratio of individual organs [25] and biochemical disturbances [26].

Also, the dose of 7.5 mg/kg 13 cRA in the animal model is equivalent to the dose of 550–600 ng/mL in humans, and 15 mg/kg 13 cRA to the 880–1650 ng/mL dose in humans [27]. The dose of 7.5 mg/kg 13 cRA applied in animals is consistent with human serum after the administration of Roaccutane capsules in acne therapy (0.5–1 mg/kg), and a dose of 15 mg/kg is twice higher than the therapeutic one in humans.

After daily administration of 13 cRA in two different concentrations for 30 days, the experiment continued for the next 30 days, without 13 cRA application, according to an already established protocol.

Anaesthesia and analgesia were applied during the animal sacrifice–the animals were adequately anesthetized by intraperitoneal administration of a mixture of 75 mg/kg of ketamine (Narketan®10, Vetoquinol AG, Belp Bern, Switzerland) and 10 mg/kg of xylazine (Xylapana® Vetoquinol Biowet Sp., Gorzow, R. Poland).

### 13-cis retinoic acid (13 cRA)

13 cRA was extracted from Roaccutane® capsules (Roche, France). Prior to the usage, 13 cRA was dissolved in sunflower oil in order to obtain concentrations of 7.5 mg/kg and 15 mg/kg. Test substances were given to rats orally via gastric tube [27].

### Body weight

Body weight was measured once a week during 60 days (9 weeks) using a digital scale to assess body mass changes in response to the diet and the 13 cRA treatment. Treatment-dependent average value was calculated for each week. The percentage of body weight change was calculated according to the formula:

Weightchange(%)=(finalweight−initialweight)100/(finalweight)

### Evaluation of food and drink consumption

Diets and the quantity of water were controlled on a daily basis. The consumption of food and drink was measured using the difference between the initial and the final weight in a 24-hour period. The results were expressed daily in grams (g) or mL.

### Adiposity index

Immediately after the rats were sacrificed, the adipose tissue fat pads were dissected and weighed. Total body fat was measured as the sum of the following individual fat pad weights: epigonadal fat + retroperitoneal fat + visceral fat. Adiposity index (%) was calculated as follows: ([*retroperitoneal fat* (*g*) + *visceral fat* (*g*) + *epigonadal fat* (*g*)] / [*body weight* (*g*)]) × 100 *and expressed as adiposity percentage* [28]. The adiposity index was used as a measure of adiposity, because the fat degree tends to increase gradually with obesity.

### Oral Glucose Tolerance Test (OGTT)

Blood glucose concentration was measured after the 60^th^ day of the experiment, after overnight fasting (8-10h), immediately after oral glucose application with gastric tube (3 g/kg) (0’ time), and continuously measured at 30´, 60´, 90´, and 120´. Blood samples for blood glucose analysis were taken from a tail vein after injection with a sterile needle. Glucose levels were measured with a glucometer (Accu-Chek Advantage II) and compatible blood glucose test strips (Roche, France). Glycaemic variation percentage within a group was calculated as a function of time (t) by applying the following formula:

%glycaemicchange=(G1−G0)x100/G1,

where G_0_ and G_1_ represent glycaemic values before the glucose treatment and at 30´, 60´, 90´, and 120´ after it, respectively.

### Haematological parameters

Blood samples for haematological analysis were taken from the abdominal aorta of each individual rat, placed in heparinized vacutainers with EDTA addition (Becton Dickinson, Plymouth, UK) and stored at 4°C till the analysis. From haematological parameters, leukocyte number (10^9^/L), differential blood count (%), erythrocytes (10^9^/L), haemoglobin (g/L), haematocrit percentage (%), and thrombocytes (10^9^/L) were analysed. Haematological parameters were determined at the Faculty of Veterinary Medicine in Zagreb using the recommended analytical methods on the Horiba ABX169 electronic counter (Micros, France).

### Biochemical parameters

Blood samples for biochemical analysis were taken from the abdominal aorta of each individual rat and collected in vacutainers without anticoagulants. Serum was used to determine the biochemical parameters using a Comprehensive Diagnostic Profile reagent rotor on a VetScan® VS2 device (Abaxis, UK). The concentration of albumin (ALB g/L), alkaline phosphatase (ALP—U/L), alanine aminotransferase (ALT—U/L), amylase (AMY—U/L), calcium (CA—mmol/L), phosphates (PHOS—mmol/L), creatinine (CRE—μmol/L), sodium (Na^+^—mmol/L), potassium (K^+^—mmol/L) and total protein (TP—g/L) were analysed.

### Serum lipids biochemical parameters and atherogenic indicators

Serum lipid levels [total cholesterol (TC), triglyceride (TG), low-density lipoprotein (LDL-c), and high-density lipoprotein (HDL-c)] were determined using Architect c 8000 (Abbott). Very-low-density lipoprotein-cholesterol (VLDL-c) concentrations were calculated as *VLDL−c* = *TG*/5. The atherogenic index (AIP), atherogenic coefficient (AC), cardiac risk (CRR), cardioprotective index (CPI) and the indicator of insulin resistance, as TG/HDL-c ratio, were calculated using the following formulas:

Atherogenic Index (AIP): *AIP* = *log* [*TG / HDL − c*]

Atherogenic Ratio (AC): *AC* = [*TG − HDL − C / HDL − c*]

Cardiac Risk Ratio (CRR): *CRR* = [*TG / HDL − c*]

Cardioprotective Index (CPI): *CPI* = [*HDL − c / LDL − c*]

An indicator of Insulin Resistance (*IR*): *IR* = *TG / HDL − c*

### Oxidative stress analyses

#### Tissue preparation

Portions of liver and kidney tissue, 75–100 mg were homogenized in 1 mL of 50 mM phosphate buffer (pH 7.0) by ultrasonic homogenizer SONOPLUS Bandelin HD2070 (Bandelin, Germany) using the MS73 probe (Bandelin, Germany) with 10% power. Homogenates were centrifuged by Micro 200R centrifuge (Hettich, Germany) for 15 minutes at a speed of 10 000 × g at +4°C. The supernatant was used for the measurements of glutathione and lipid peroxidation level, as well as catalase (CAT) activity. All methods are described in previously published work by Brzovic Saric et al. [29].

All parameters normalized in relation to exact protein content.

#### Lipid peroxidation

The presence of lipid peroxidation was determined by measuring the concentration of malondialdehyde (MDA), the major products of lipid peroxidation. Malondialdehyde reacts with thiobarbituric acid and produces a chromogen which can be measured spectrophotometrically. A total of 100 μL supernatant was mixed with 100 μL of 8.1% aqueous SDS, 750 μL of 20% acetic acid (pH = 3.5) and 750 μL of 0.81% aqueous thiobarbituric acid. The mixture was heated for 60 minutes at a temperature of 95°C. After the cooling of the samples on ice, the absorbance (A) was measured at 532 nm with Libra S [spectrophotometer (Biochrom, UK). The total MDA concentration (c) was calculated using the extinction coefficient for MDA (ε = 1.56 x 10^5^ M^-1^) according to the following formula:

c=(A_sample×V_(reactionmixtures)(mL))/(ε×V_(sample(mL))×c_(proteininthesample(mg/mL))).

The total concentration of MDA is expressed as nmol MDA per mg of protein. The concentration of lipid peroxides was expressed as mg MDA/mL/mg protein measured by the Lowry method [30].

#### Glutathione assay

The glutathione assay is described before [27]. In short, 200 μL of 3 mM of 5–5'-dithiobis [2-nitrobenzoic acid] (DTNB, Ellman's Reagent) was added to 30 μL of sample supernatant. DTNB reacts with GSH to form chromospheres, 5-thionitrobenzoic acid (TNB) and GSTNB. The absorbance was measured at 412 nm. The results were calculated from the standard curve of reduced glutathione measured by the same protocol.

#### Catalase (CAT) activity

Catalase activity was determined using the spectrophotometric method described previously [31]. In this method, catalase activity was estimated by a decrease in absorbance of H_2_O_2_ at 240 nm. The reaction mixture of total volume of 1 mL contained 980 μL 10 mM H_2_O_2_ (in phosphate buffer, pH 7.0) and 20 μL PBS or a tissue sample. Catalase activity was measured by the extinction coefficient of H_2_O_2_ (ε = 39.4 mM^-1^cm^-1^); the specific activity was calculated and expressed as μmoles/min/mg of total protein.

### Statistical analysis

The data was presented as mean ± standard error (SEM) of the representative experiment from three independent experiments. All data was analysed by Kruskal-Wallis ANOVA. Further analysis of the differences between the groups was made with multiple comparisons of mean ranks for all groups. Statistical analyses were performed using STATISTICA 12 software (StatSoft, Tulsa, OK, USA). The data was considered significant at *P* < 0.05.

## Results

### Effect of 13 cRA on body weight change in rats fed standard or high fat diets

**Fig 1:**
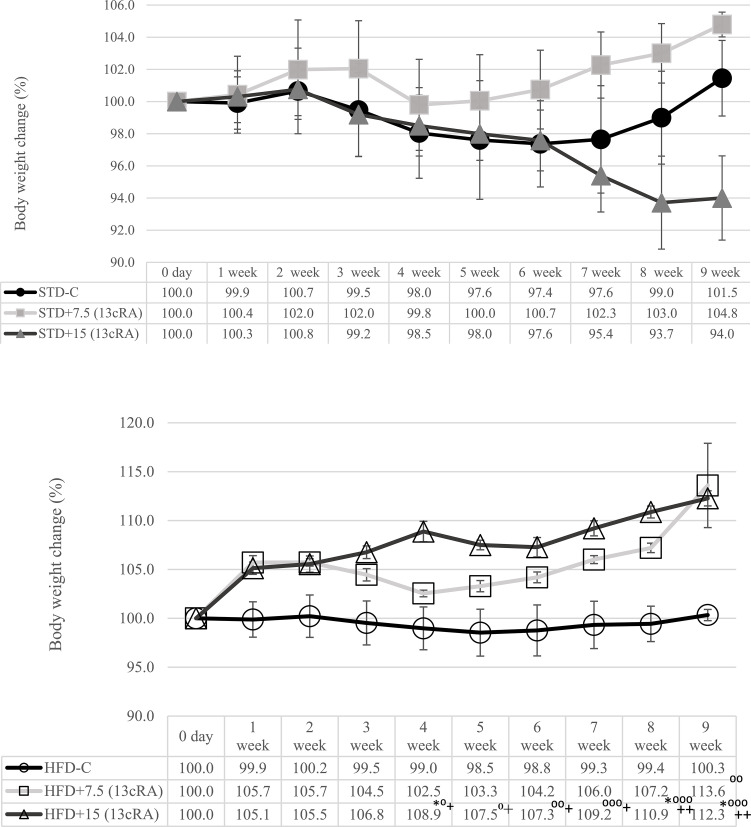
Effect of 13 cRA on body weight change in rats fed standard or high fat diets. Changes in body weight during the experiment at different time points. Before the beginning of the experiment, animals were weighed and grouped with regards to their body weight. Weights were measured once a week. The percentage change in weight was calculated for each group as follows: *Percentage change in weight* = (*Final weight − Initial weight*) *x* 100 / *Final weight*. Rats were fed either STD or HFD for 9 weeks *ad libitum*. 13 cRA was orally administered to rats via a gastric tube in two different concentrations (7.5 mg/kg, 15 mg/kg) during 30 days. Number of rats per group: 6. Data is expressed as mean ± SEM. Data was analysed using Kruskal-Wallis ANOVA (STATISTICA 13 StatSoft, Tulsa, OK, USA). *Different in relation to STD-C (* *P*≤0.05). +Different in relation to HFD-C (+ *P*≤0.05; ++ *P*≤0.01). ^⁰^Different in relation to STD+15 RA (^⁰^ *P*≤0.05, ^⁰⁰^ *P*≤0.01, ^⁰⁰⁰^ *P*≤0.001).

With the aim of investigating whether 13 cRA affects the metabolism of rats fed STD or HFD diet, rats received 13 cRA orally using a gastric cannula at a dose of 7.5 or 15 mg/kg for 30 days. Percentage of the animal body weight change during the experiment is shown in Fig 1. Animals fed HFD and 13 cRA administered in two different concentrations (7.5 mg/kg, 15 mg/kg) reached the highest body weights at the end the experiment. The weight increase in relation to the intial body weight in the 13 cRA-treated group at both doses (7.5 and 15 mg/kg) on HFD diet was 13.6 or 12.3% (Fig 1B), while STD-fed animals with the presence of 13 cRA showed a 6% weight loss at 15 mg/kg 13 cRA (Fig 1A).

### Nutritional parameters, fat pad mass and adiposity index

The results of nutritional parameters (food and drink intake), fat pad mass and adiposity index are shown in Table 2. We observed that the animals of the control group, fed with the standard diet, consumed more food and drink in comparison to the other groups, with no differences during the treatment period (Table 2). We also noticed that the HFD rats which were administered 13 cRA consumed more food in comparison to the animals that consumed HFD alone, with no significant difference (*P* >0.05). HFD+15 (13 cRA) increased visceral, retroperitoneal and perigonadal fat pad mass, total body fat or adiposity index compared to STD+15 (13 cRA) (*P*<0.01, *P*<0.001) (Table 2). A difference in adiposity index was also noticed in HFD+7.5 (13 cRA) group compared to the STD+15 (13 cRA) group (*P<*0.01) (Table 2).

**Table 2:** Nutritional parameters, fat pad mass and adiposity index.

Group^a^	Mean food intake (g/day)	Drink ingested (mL/day)	Perigonadal fat (% BW)	Retroperitoneal fat (% BW)	Visceral fat (% BW)	Adiposity index (%)
STD-C	21.69 ± 1.43	36.03 ± 1.19	1.04 ± 0.15	1.09 ± 0.07	0.99 ± 0.11	3.12 ± 0.03
STD+7.5 (13 cRA)	19.95 ± 1.38	35.76 ± 1.9	0.81 ± 0.03	0.85 ± 0.06	0.77 ± 0.04	2.43 ± 0.02
STD+15 (13 cRA)	16.94 ± 1.70	34.92 ± 1.1	0.72 ± 0.01	0.75 ± 0.04	0.68 ± 0.02	2.15 ± 0.02
HFD-C	18.45 ± 1.31	28.99 ± 1.35	1.28 ± 0.05	1.45 ± 0.23	1.39 ± 0.07	4.12 ± 0.05
HFD+7.5 (13 cRA)	19.73 ± 2.07	30.37 ± 0.85	1.37 ± 0.01	1.55 ± 0.11	1.49 ± 0.03	4.41 ± 0.05°
HFD+15 (13 cRA)	21.90 ± 1.80	30.02 ± 0.69	1.52 ± 0.02°	1.72 ± 0.03°	1.65 ± 0.06°	4.89 ± 0.06*°°°

^a^Rats were fed either STD or HFD for 9 weeks *ad libitum*. 13 cRA was orally administered to rats via a gastric tube in two different concentrations (7.5 mg/kg, 15 mg/kg) during 30 days. Visceral, perigonadal and retroperitoneal adipose tissue was removed and weighed during animal sacrifice. Adiposity index (%) was calculated as: ([retroperitoneal fat (g) + visceral fat (g) + epigonadal fat (g)] / [body weight (g)]) × 100 and expressed as adiposity percentage. Number of rats per group: 6.

*Statistically significantly different in relation to STD-C (* *P*≤0.05). ^⁰^Statistically significantly different in relation to STD+15 RA (^⁰⁰^ *P*≤0.01, ^⁰⁰⁰^ P<0.001). Data are expressed as mean ± SEM. Data were analysed using Kruskal-Wallis ANOVA (STATISTICA 13 StatSoft, Tulsa, OK, USA).

### Effect of 13 cRA on leptin and adiponectin levels in rats fed standard or high fat diets

The excess fat mass that characterizes obesity is produced by an expansion of adipose tissue not only as an inert energy reservoir, but also as an endocrine organ producing various adipokines such as leptin, adiponectin, adipsin, resistin, and approximately 50 biologically active proteins. Adiponectin and leptin are the most abundant peptides secreted by adipocytes, and play a central role in obesity-related diseases. In order to understand whether HFD alone and in combination with 13 cRA has an effect on obesity and weight control, we investigated leptin and adiponectin levels and lipid parameters (triglyceride [TG] and total cholesterol levels [TC], HDL-c, LDL-c).

**Fig 2:**
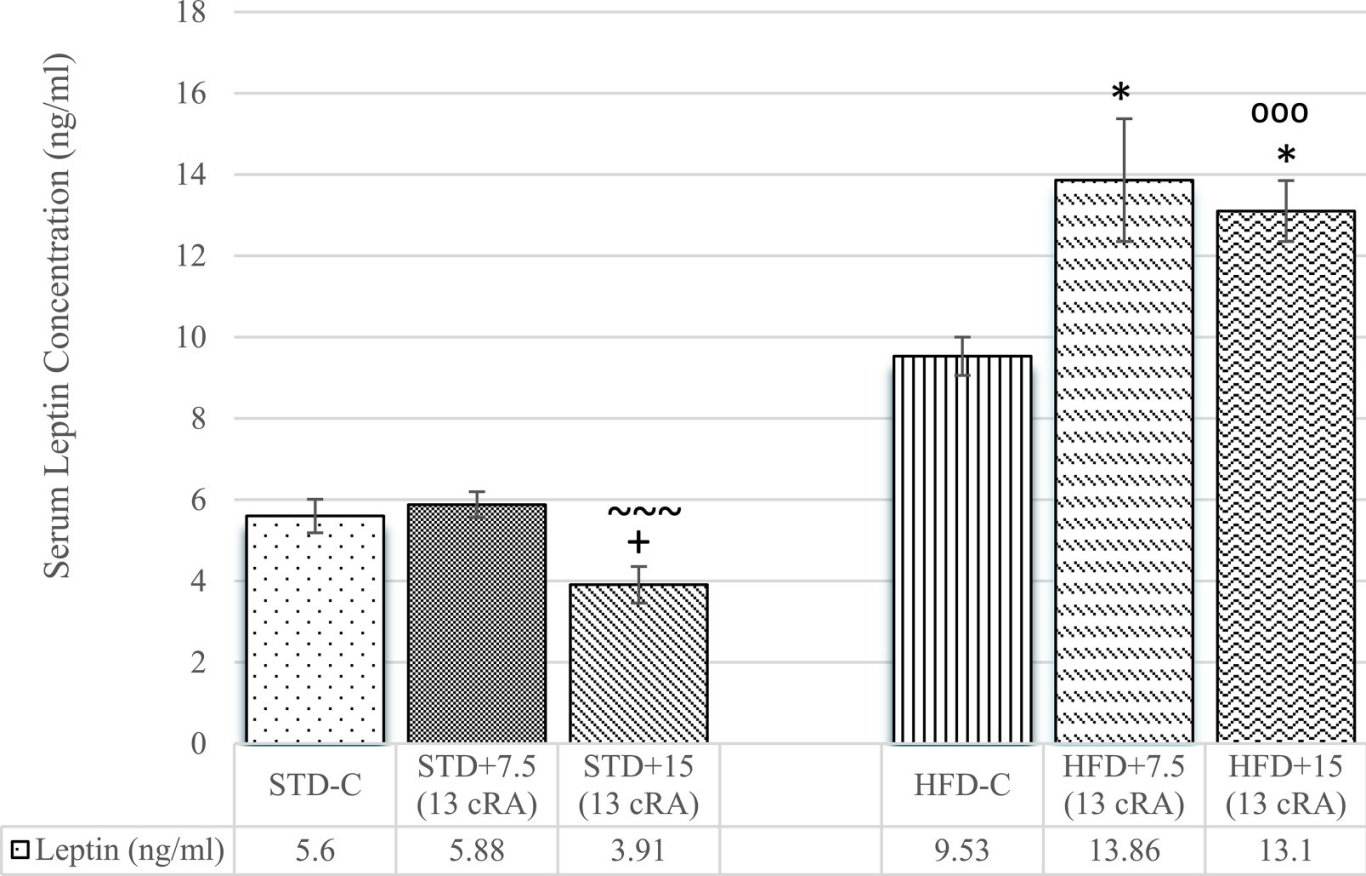
Effect of 13 cRA on leptin level in rats fed standard or high fat diets. Rats were fed either STD or HFD for 9 weeks *ad libitum*. 13 cRA was orally administered to rats via a gastric tube in two different concentrations (7.5 mg/kg, 15 mg/kg) during 30 days. Blood for leptin analysis was taken from abdominal aorta at the end of the experiment in morning hours. Number of rats per group: 6. *Different in relation to STD-C (* *P*≤0.05). ~Different in relation to HFD+7.5 (13 cRA) (~~~ *P*≤0.001). +Different in relation to HFD-C (+ *P*≤0.05). ^⁰^Different in relation to STD+15 (13 cRA) (^⁰⁰⁰^ *P*≤0.001). Data is expressed as mean ± SEM. Data was analysed using Kruskal-Wallis ANOVA (STATISTICA 13 StatSoft, Tulsa, OK, USA).

Leptin levels are shown in Fig 2. Increase in leptin levels was noticed in experimental groups fed HFD with 13 cRA (7.5 mg/kg and 15 mg/kg) added in comparison to the control group fed STD (STD-C; *P*<0.05) (Fig 2). Slight increase was also seen in HFD-C, with no significant difference. The lowest leptin levels were noticed in STD+15 (13 cRA), which was significant in comparison to HFD-C (*P*<0.05) and HFD+7.5 (13 cRA) (*P*<0.001).

**Fig 3:**
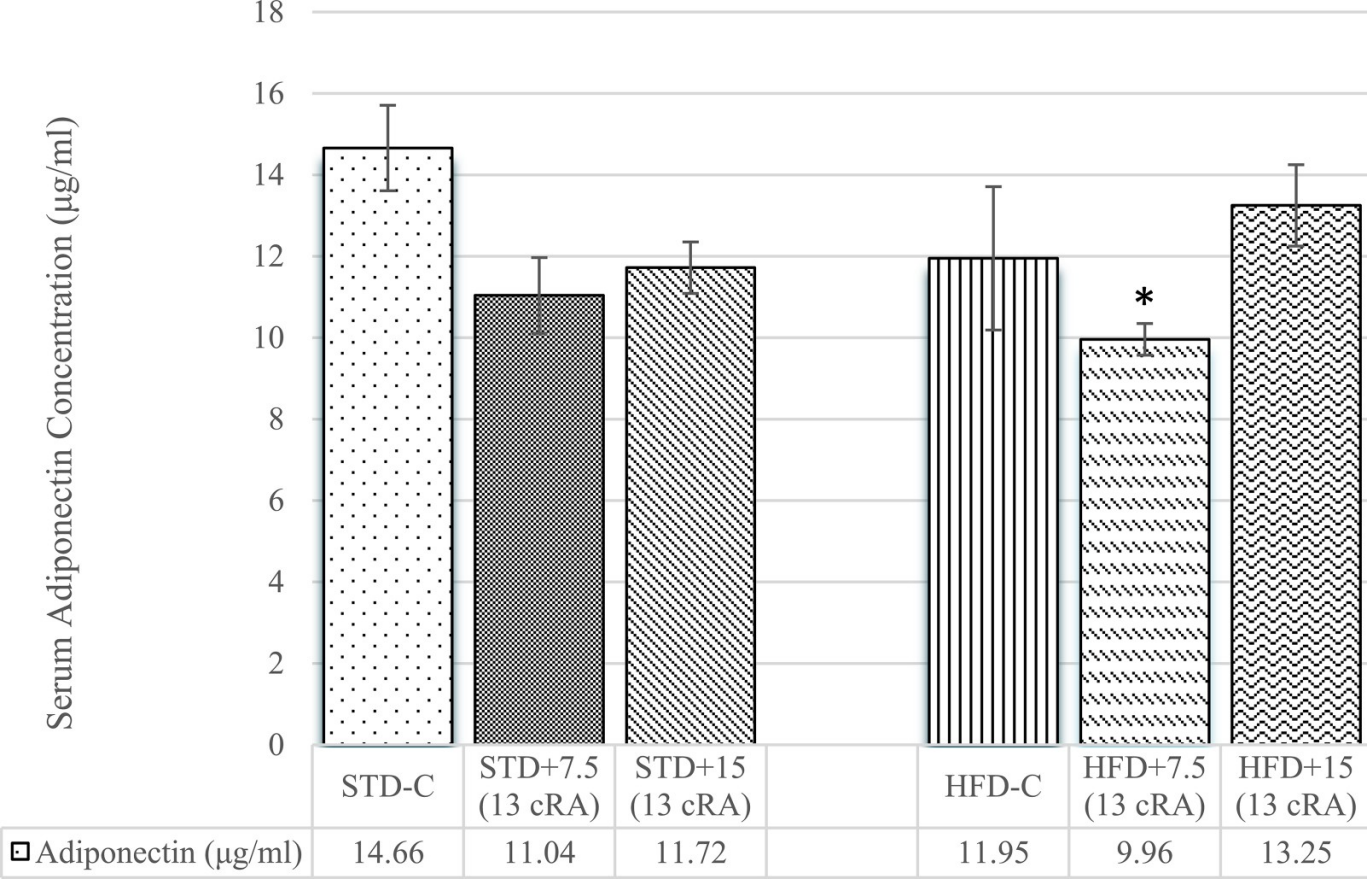
Effect of 13 cRA on adiponectin level in rats fed standard or high fat diets. Rats were fed either STD or HFD for 9 weeks *ad libitum*. 13 cRA was orally administered to rats via a gastric tube in two different concentrations (7.5 mg/kg, 15 mg/kg) during 30 days. Blood for adiponectin analysis was taken from abdominal aorta at the end of the experiment in morning hours. Number of rats per group: 6. *Different in relation to STD-C (*P≤0.05). Data are expressed as mean ± SEM. Data were analysed using Kruskal-Wallis ANOVA (STATISTICA 13 StatSoft, Tulsa, OK, USA).

Adiponectin levels are shown in Fig 3. The lowest adiponectin concentration was seen in HFD+7.5 (13 cRA), with difference in comparison to STD-C (*P*<0.05).

### Effect of 13 cRA on serum lipid biochemical parameters and atherogenic indicators in rats fed standard or high fat diets

TC, TG, HDL-c, and LDL-c concentrations in serum were analysed to observe the dyslipidaemic effect of 13 cRA in STD or HFD fed rats. Lipidogram is shown in Table 3.

**Table 3:** Effect of 13 cRA on serum lipid biochemical parameters in rats fed standard or high fat diets.

	SERUM LIPID BIOCHEMICAL PARAMETERS (X ± SEM)				
Group^a^	TC (mmol/L)	TG (mmol/L)	HDL-c (mmol/L)	LDL-c (mmol/L)	VLDL-c (mmol/L)
**STD-C**	1.27 ± 0.02	0.53 ± 0.04	0.49 ± 0.04	0.4 ± 0.05	0.10 ± 0.02
**STD+7.5 (13 cRA)**	1.33 ± 0.01	0.81 ± 0.01	0.38 ± 0.02^+^	0.5 ± 0.03	0.16 ± 0.01
**STD+15 (13 cRA)**	1.3 ± 0.02^~^	1.04 ± 0.04	0.38 ± 0.02^++^	0.59 ± 0.04	0.20 ± 0.01
**HFD-C**	1.45 ± 0.01	1.12 ± 0.02	0.2 ± 0.02***	0.8 ± 0.03*	0.22 ± 0.03
**HFD+7.5 (13 cRA)**	1.51 ± 0.03**	1.41 ± 0.06***	0.28 ± 0.01*	0.85 ± 0.05**^x^	0.28 ± 0.02*
**HFD+15 (13 cRA)**	1.57 ± 0.02***^xππ^	1.9 ± 0.01***^xxπ^	0.31 ± 0.02	1 ± 0.09***^xx^	0.38 ± 0.04**

^a^Rats were fed either STD or HFD for 9 weeks *ad libitum*. 13 cRA was orally administered to rats via a gastric tube in two different concentrations (7.5 mg/kg, 15 mg/kg) during 30 days. Blood for plasma lipid analysis was taken from abdominal aorta at the end of the experiment. Number of rats per group: 6.

*Different in relation to STD-C (*P≤0.05, **P≤0.01, ***P≤0.001). ^+^Different in relation to HFD-C (^+^*P*≤0.05, ^++^*P*≤0.01). ^x^Different in relation to STD+7.5 (13 cRA) (^x^*P*≤0.05, ^xx^*P*≤0.01). ^π^Different in relation to STD+15 (13 cRA) (^π^*P*≤0.05, ^ππ^*P*≤0.01). Data are expressed as mean ± SEM. Data were analysed using Kruskal-Wallis ANOVA (STATISTICA 13 StatSoft, Tulsa, OK, USA). Abbreviations: TC–total cholesterol, TG—triglyceride, HDL-c–high density lipoproteins, LDL-c–low density lipoproteins, VLDL-c–very low density lipoproteins.

VLDL-c levels showed an increase in animals fed HFD with 13 cRA added in both concentrations in comparison to STD-C (*P*<0.05, *P*<0.01). An increase in LDL-c levels was seen among animals fed HFD, with difference in all groups in comparison to STD-C (*P*<0.05, *P*<0.01, *P*<0.001). Animals fed STD with added 13 cRA showed a slight increase in LDL-c levels, but with no difference. HDL-c was lower in animals fed HFD, with difference in HFD-C and HFD+7.5 (13 cRA) in comparison to STD-C (*P*<0.001, *P*<0.05). 13 cRA administration in rats fed STD also caused a decrease in HDL levels, but with no significant difference in comparison to STD-C. TC was higher in HFD groups, both in control and experimental groups, with an increase in HFD+7.5 (13 cRA) and HFD+15 (13 cRA) in comparison to STD-C (*P*<0.01, *P*<0.001). 13 cRA administration in STD fed animals also caused a slight increase in TC levels, with no difference. TG levels increased depending of the dose of 13 cRA administered in HFD groups as well, with a difference in HFD+7.5 (13 cRA) and HFD+15 (13 cRA) in comparison to STD-C (*P*<0.001).

**Fig 4:**
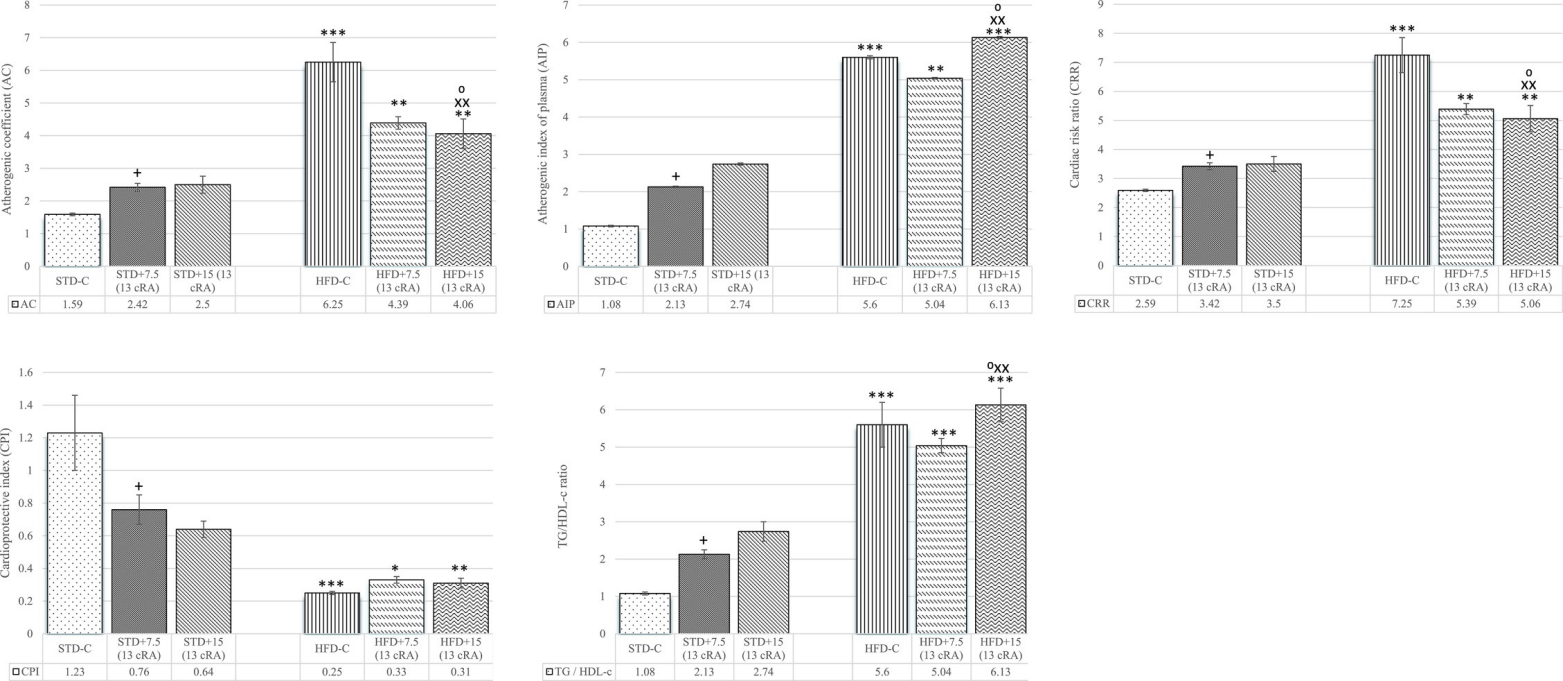
Effect of 13 cRA on atherogenic indicators in rats fed standard or high fat diets. Rats were fed either STD or HFD for 9 weeks *ad libitum*. 13 cRA was orally administered to rats via a gastric tube in two different concentrations (7.5 mg/kg, 15 mg/kg) during 30 days. Blood for plasma lipids analysis was taken from abdominal aorta at the end of the experiment. Number of rats per group: 6. *Different in relation to STD-C (*P £ 0.05; **P £ 0.01; ***P £ 0.001). xDifferent in relation to STD+7.5 (13 cRA) (x*P* £ 0.05; xx*P* £ 0.01). +Different in relation to HFD-C (+*P* £ 0.05). ^⁰^Different in relation to STD+15 (13 cRA) (^⁰^*P* £ 0.05). Data are expressed as mean ± SEM. Data were analysed using Kruskal-Wallis ANOVA (STATISTICA 13 StatSoft, Tulsa, OK, USA).

AC, AIP, CRR, CPI, and TG/HDL-c ratio (Fig 4A–4E) were calculated from serum lipid biochemical parameters.

An increase in AC, AIP and CRR was noticed among animals fed HFD (Fig 4A–4C). AC was 3.93 times higher in HFD-C (*P*<0.001), 2.76 times in HFD+7.5 (13 cRA) (*P*<0.01) and 2.55 times in HFD+15 (13 cRA) (*P*<0.01) in comparison to STD-C. AIP was 5.18 times higher in HFD+15 (13 cRA) (*P*<0.001). An increase was also seen in HFD-C (*P*<0.001) and HFD+7.5 (13 cRA) (*P*<0.01) in comparison to STD-C. The highest CRR was found in HFD-C, with a significant difference in comparison to STD-C (*P*<0.001). Marker of insulin resistance, which is a TG/HDL-c ratio, is shown in Fig 4E. An increase was seen in HFD-C, HFD+7.5 (13 cRA) and HFD+15 (13 cRA) in comparison to STD-C (*P*<0.001). On the other hand, the lowest values of CPI were present in groups fed HFD (Fig 4D). CPI of HFD-C was 4.92 times lower in comparison to STD-C (*P*<0.001). A decrease was also observed in HFD+7.5 (13 cRA) (*P*<0.05) and HFD+15 (13 cRA) (*P*<0.01).

### Effect of 13 cRA on oral glucose tolerance test (OGTT) in rats fed standard or high fat diets

**Fig 5:**
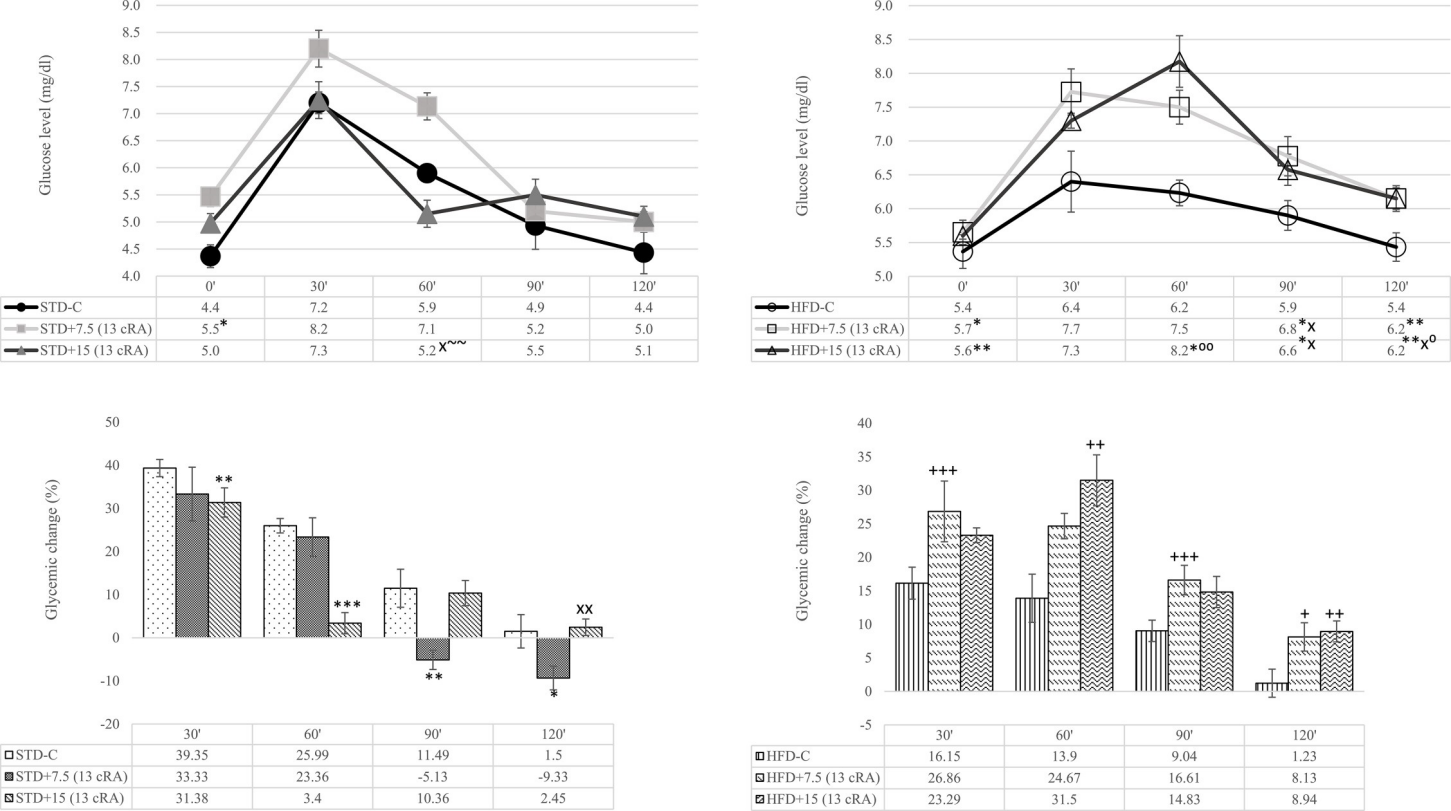
Effect of 13 cRA on oral glucose tolerance test-OGTT (A, B) and the percentage of glycaemic variation in rats fed standard (C) or high fat diets (D). Rats were fed either STD or HFD for 9 weeks *ad libitum*. 13 cRA was orally administered to rats via a gastric tube in two different concentrations (7.5 mg/kg, 15 mg/kg) during 30 days. Glucose solution (30%) was given to rats orally via gastric tube on the 60^th^ day after overnight (8–10 h) fasting and glucose was measured at 0´, 30´, 60´, 90´, 120´. Percentage of glycaemic variation within a group was calculated as a function of time (t) by applying the following formula: % *glycaemic change* = (*G*1 − *G*0) *x*100/*G*1, where G_0_ and G_1_ represent glycaemic values before the glucose treatment and at 30´, 60´, 90´, 120´ after the glucose treatment, respectively. Number of rats per group: 6. *Different in relation to STD-C (* *P*≤0.05, ** *P*≤0.01, *** *P*≤0.001). xDifferent in relation to STD+7.5 (13 cRA) (x *P*≤0.05, xx *P*≤0.01). +Different in relation to HFD-C (+*P*≤0.05, ++ *P*≤0.01, +++ *P*≤0.001). ~Different in relation to HFD+7.5 (13 cRA) (~~ *P*≤0.01). ^⁰^Different in relation to STD+15 (13 cRA) (^⁰^*P*≤0.05, ^⁰⁰^ *P*≤0.01). Data are expressed as mean ± SEM. Data were analysed using Kruskal-Wallis ANOVA (STATISTICA 13 StatSoft, Tulsa, OK, USA).

OGTT is the most widely used test for assessing glucose homeostasis in rodents where overnight fasting increases insulin sensitivity [32]. Higher glucose levels were observed at the onset of OGTT (0′) in experimental groups administered 13 cRA, both in STD and HFD groups (STD+7.5 (13 cRA), *P*<0.05) (HFD+7.5 (13 cRA), *P*<0.05) (HFD+15 (13 cRA), *P*<0.01). At the end of the test (120'), there was an increase in glucose concentration in HFD+7.5 (13 cRA) group (*P*<0.01) and HFD+15 (13 cRA) (*P*<0.01) compared to the control group (STD–C), suggesting that the treatment with 13 cRA and HFD results in higher glucose concentrations and influences glucose metabolism (Fig 5A and 5B). The percentage of glycaemic variation within a group after oral glucose application to rats administered 13 cRA in combination with STD or HFD was calculated as a function of time and is shown in Fig 5C and 5D. Interestingly, 13 cRA shows completely opposite effects on glycaemic changes in rats fed STD compared to the HFD diet.

### Effect of 13 cRA on haematological and biochemical parameters in rats fed standard or high fat diets

Results in Table 4 indicate that 13 cRA and HFD caused an increase in the total leukocyte count (*P*<0.01, *P*<0.001) in relation to STD+7.5 (13 cRA) and HFD+15 (13 cRA). Differential blood analysis showed increased neutrophil counts in the HFD+15 (13 cRA) compared to the control (*P*<0.05) and STD+15 (13 cRA) (*P*<0.001) (Table 4).

**Table 4:** Effect of 13 cRA on haematological parameters in rats fed standard or high fat diets.

HAEMATOLOGICAL PARAMETERS (X ± SEM)							
Group^a^	Erythrocytes (x10^12^/L)	Haemoglobin (g/L)	Haematocrit (%)	Thrombocytes (x 10^9^/L)	Total leukocytes (x10^9^/L)	Segmented neutrophils (%)	Lymphocytes (%)
**STD-C**	7.82 ± 0.26	150.33 ± 3.67	41.33 ± 1.28	582 ± 20.78	6.13 ± 0.05	19.33 ± 2.74	70.67 ± 6.02
**STD+7.5 (13 cRA)**	7.25 ± 0.06	141.5 ± 0.28	38.5 ± 0.28	624.5 ± 4.33	6 ± 0.07	18.5 ± 3.75	79.5 ± 4.33
**STD+15 (13 cRA)**	7.25 ± 0.09	141.33 ± 0.91^++^	38.33 ± 0.55^+^	693 ± 18.43^~~^	6 ± 0.03^~^	14 ± 2.39	86 ± 2.39
**HFD-C**	7.88 ± 0.09	156.67 ± 2.55	42 ± 0.44	674.33 ± 3.83	7.48 ± 0.2	25 ± 1.59	74 ± 1.31
**HFD+7.5 (13 cRA)**	7.67 ± 0.23	150 ± 3.18	40.67 ± 1.28	563.33 ± 17.34^++^	7.6 ± 0.19^X^	25 ± 1.31	74.67 ± 1.38
**HFD+15 (13 cRA)**	7.6 ± 0.03	152 ± 0.73	41 ± 0.36	568 ± 5.87^++π^	7.82 ± 0.12^Xππ^	36.33 ± 2.95*^πππ^	77.33 ± 2.74

^a^Rats were fed either STD or HFD for 9 weeks *ad libitum*. 13 cRA was orally administered to rats via a gastric tube in two different concentrations (7.5 mg/kg, 15 mg/kg) during 30 days. Blood for haematological analysis was taken from abdominal aorta. Number of rats per group: 6.

*Different in relation to STD-C (*P<0.05). ^+^Different in relation to HFD-C (^+^*P*<0.05; ^++^*P*<0.01). ^x^Different in relation to STD+7.5 RA (^x^*P*<0.05). ~Different in relation to HFD+7.5 RA (^~^*P*<0.05; ^~~^*P*<0.01). ^π^Different in relation to HFD+15 RA (^π^*P*<0.05; ^ππ^*P*<0.01; ^πππ^*P*<0.001). Data are expressed as mean ± SEM. Data were analysed using Kruskal-Wallis ANOVA (STATISTICA 13 StatSoft, Tulsa, OK, USA).

Biochemical parameters are shown in Table 5. Albumin levels decreased in STD+7.5 (13 cRA) (*P*<0.01) and STD+15 (13 cRA) (*P*<0.01) compared to the control group (STD-C). A decrease in albumin levels was also seen in the HFD group, with a difference in HFD+7.5 (13 cRA) compared to STD+7.5 (13 cRA) (*P*<0.05). Treatment with 13 cRA resulted in an increase in ALP levels both in the STD, as well as in the HFD group, with no difference in the STD group. Amylase increased in animals fed HFD depending on the 13 cRA application, with an increase in HFD+15 (13 cRA) in comparison to STD-C (*P*<0.01) and STD+7.5 (13 cRA) (*P*<0.01). Calcium levels increased in HFD+7.5 (13 cRA) and HFD+15 (13 cRA) compared to STD-C (*P*<0.01; *P*<0.001). Potassium levels decreased in HFD+15 (13 cRA) compared to STD-C (*P*<0.001). Total proteins decreased in the HFD group, with a difference in HFD+15 (13 cRA) compared to STD-C (*P*<0.05). Creatinine levels increased depending on the dose of RA applied, but with no significant difference (Table 5).

**Table 5:** Effect of 13 cRA on biochemical parameters in rats fed standard or high fat diets.

BIOCHEMICAL PARAMETERS (X ± SEM)									
Group^a^	ALB (g/L)	ALP (U/L)	AMY (U/L)	Ca (mmol/L)	PHOS (mmol/L)	Na+ (mmol/L)	K+ (mmol/L)	TP (g/L)	CRE (umol/L)
**STD-C**	53 ± 1.28	181 ± 9.31	576.17 ± 32.83	2.41 ± 0.01	1.92 ± 0.36	134 ± 0.63	5.43 ± 0.16	67 ± 0.55	32.33 ± 3.11
**STD+7.5 (13 cRA)**	45.33 ± 1.26**^+^	204.17 ± 20.73	583.5 ± 51.09	2.45 ± 0.12	1.84 ± 0.36	134.67 ± 2.07	4.77 ± 0.75	67.83 ± 3.39	37 ± 2.38
**STD+15 (13 cRA)**	44.33 ± 1.05**^+^^~^	210 ± 15.81	613.67 ± 37.45	2.52 ± 0.04	1.79 ± 0.15	132.67 ± 0.76	4.53 ± 0.14	64.33 ± 1.05	39 ± 5.07
**HFD-C**	52.33 ± 1.52	177.83 ± 15.85	616.33 ± 4.83	2.54 ± 0.02	1.94 ± 0.03	131.67 ± 0.21	4.77 ± 0.2	65.67 ± 1.15	32.33 ± 1.73
**HFD+7.5 (13 cRA)**	51 ± 1.17^x^	178.75 ± 11.67^x^	634.25 ± 2.45	2.57 ± 0.01**	1.89 ± 0.06	131.25 ± 0.31*^x^	4.7 ± 0.07	64.75 ± 1.17	35.25 ± 2.16
**HFD+15 (13 cRA)**	51.13 ± 0.31	204 ± 4.51	654.25 ± 11.38**^xx^	2.82 ± 0.1***^xx^	1.91 ± 0.05	132 ± 0.37	4.4 ± 0.11***	59.75 ± 0.49*^xx^	36.25 ± 3.34

^a^Rats were fed either STD or HFD for 9 weeks *ad libitum*. 13 cRA was orally administered to rats via a gastric tube in two different concentrations (7.5 mg/kg, 15 mg/kg) during 30 days. Blood for biochemical analysis was taken from abdominal aorta. Number of rats per group: 6.

*Different in relation to STD-C (*P<0.05; **P<0.01; ***P<0.001).

^+^Different in relation to HFD-C (^+^*P*<0.05). ^x^Different in relation to STD+7.5 (13 cRA) (^x^*P*<0.05; ^xx^*P*<0.01). ^~^Different in relation to HFD+7.5 (13 cRA) (^~^*P*<0.05). Data are expressed as mean ± SEM. Data were analysed using Kruskal-Wallis ANOVA (STATISTICA 13 StatSoft, Tulsa, OK, USA). Abbreviation: ALB–Albumins, ALP–Alkaline phosphatase, AMY–Amylase, Ca–Calcium, PHOS–Phosphate, Na+–Natrium, K+–Potassium, TP–Total Proteins, CRE–Creatinine

**Fig 6:**
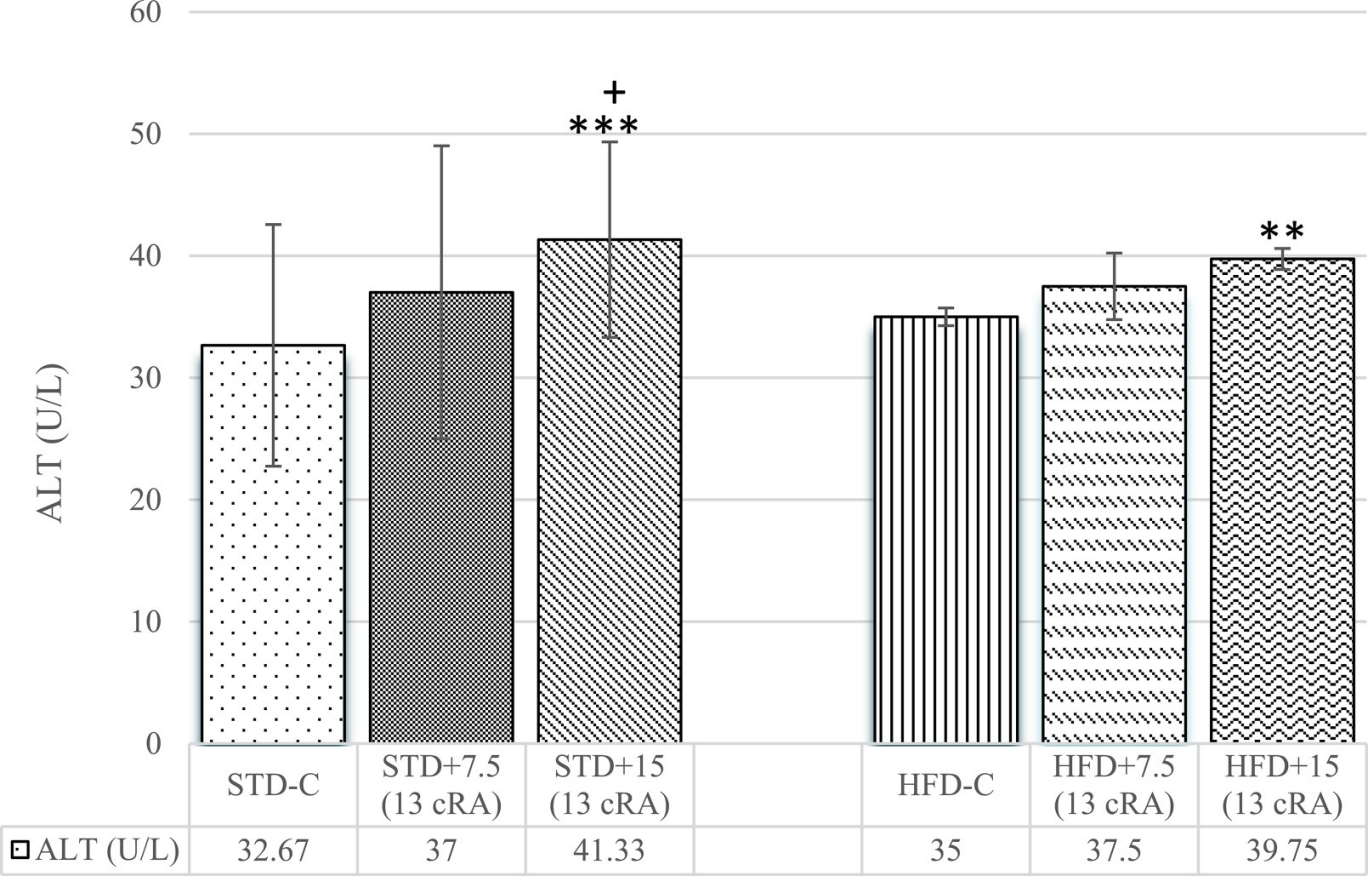
Effect of 13 cRA on ALT in rats fed standard or high fat diets. Rats were fed either STD or HFD for 9 weeks *ad libitum*. 13 cRA was orally administered to rats via a gastric tube in two different concentrations (7.5 mg/kg, 15 mg/kg) during 30 days. Blood for biochemical analysis was taken from abdominal aorta. Number of rats per group: 6. *Different in relation to STD-C (**P<0.01; ***P<0.001). ^+^Different in relation to HFD-C (^+^*P*<0.05). Data are expressed as mean ± SEM. Data were analysed using Kruskal-Wallis ANOVA (STATISTICA 13 StatSoft, Tulsa, OK, USA). Abbreviation: ALT–Alanine Aminotransferase.

An increase in ALT levels was noticed both for STD, as well as HFD, depending on the dose of administered 13 cRA (Fig 6). Difference was observed in STD+15 (13 cRA) (*P*<0.001) and HFD+15 (13 cRA) (*P*<0.01) in comparison to the control group (STD-C).

**Fig 7:**
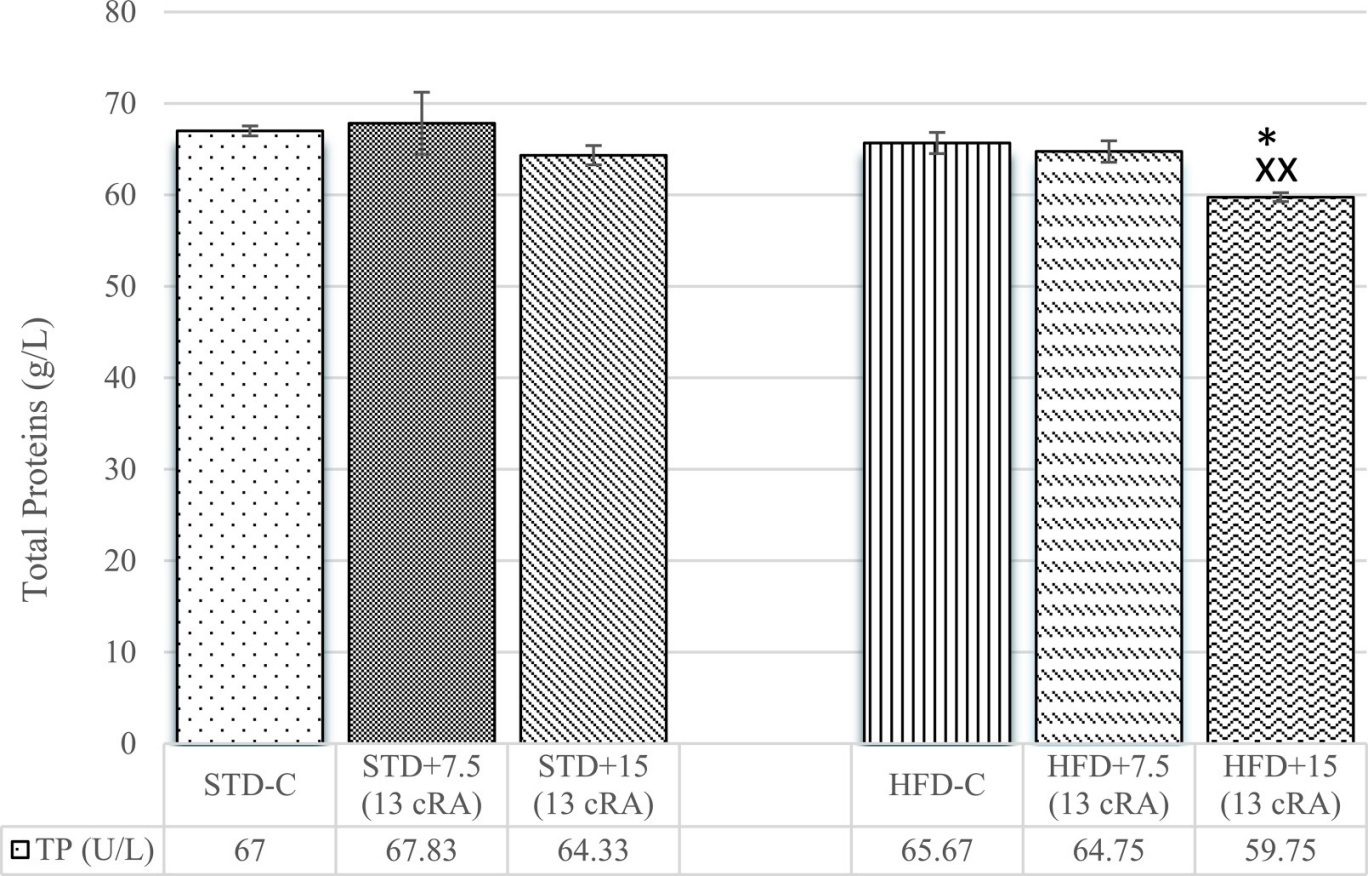
Effect of 13 cRA on total protein level in rats fed standard or high fat diets. Rats were fed either STD or HFD for 9 weeks *ad libitum*. 13 cRA was orally administered to rats via a gastric tube in two different concentrations (7.5 mg/kg, 15 mg/kg) during 30 days. Blood for biochemical analysis was taken from abdominal aorta. Number of rats per group: 6. *Different in relation to STD-C (*P<0.05). ^x^Different in relation to STD+7.5 (13 cRA) (^xx^*P*<0.01). Data are expressed as mean ± SEM. Data were analysed using Kruskal-Wallis ANOVA (STATISTICA 13 StatSoft, Tulsa, OK, USA). Abbreviation: TP–Total Proteins.

A decrease in total protein levels was seen in HFD+15 (13 cRA) in comparison to STD-C (*P*<0.05) and STD+7.5 (13 cRA) (*P*<0.01) (Fig 7).

### Effect of 13 cRA on oxidative stress parameters in liver and kidney tissues in rats fed standard or high fat diets

Irregular production of adipokines in obesity induces the production of ROS, but the relationship between the changes in adipokine levels and oxidative stress remains unclear. In order to study the relationship between blood adipokine levels and oxidative stress markers, we investigated the effect of 13 cRA on sensory markers of kidney and liver oxidative stress of STD or HFD fed rats.

**Fig 8:**
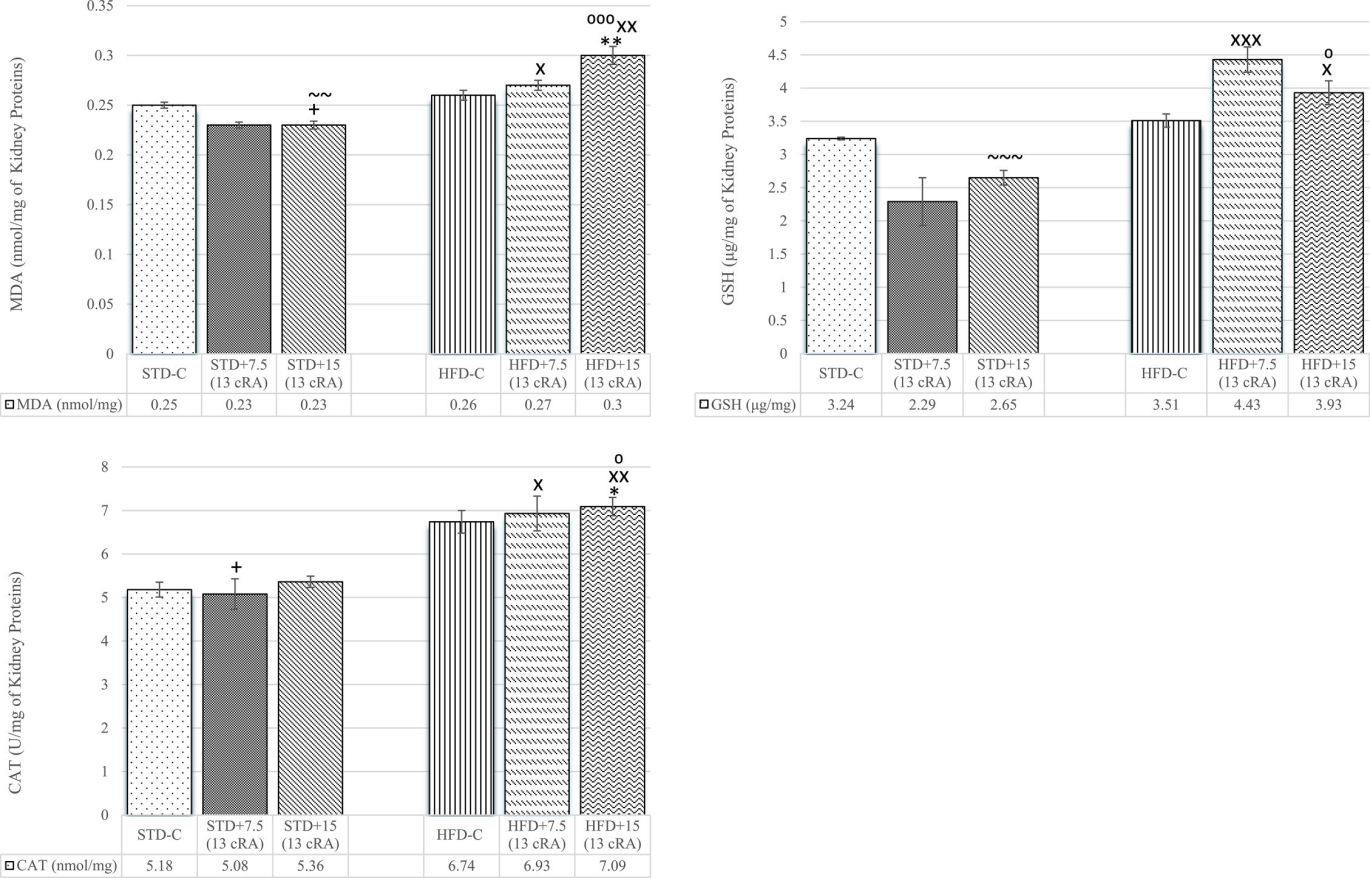
Effect of 13 cRA on MDA (A), GSH (B) and kidney CAT activity (C) of rats fed standard or high fat diets. Rats were fed either STD or HFD for 9 weeks *ad libitum*. 13 cRA was orally administered to rats via a gastric tube in two different concentrations (7.5 mg/kg, 15 mg/kg) during 30 days. Kidneys were isolated when animals were sacrificed, on the 60^th^ day of the experiment. Number of rats per group: 6. *Different in relation to STD-C (* *P*≤0.05; ** *P*≤0.01). +Different in relation to HFD-C (+ *P*≤0.05). XDifferent in relation to STD+7.5 (13 cRA) (x *P*≤0.05; xx *P*≤0.01; xxx *P*≤0.001). ^⁰^Different in relation to STD+15 (13 cRA) (^⁰^ *P*≤0.05; ^⁰⁰⁰^ *P*≤0.001). ~Different in relation to HFD+7.5 (13 cRA) (~~ *P*≤0.01; ~~~ *P*≤0.001). Data are expressed as mean ± SEM. Data were analysed using Kruskal-Wallis ANOVA (STATISTICA 13 StatSoft, Tulsa, OK, USA).

An increase in kidney MDA levels was observed in HFD+7.5 (13 cRA) in comparison to STD+7.5 (13 cRA) (*P*<0.05), as well as in HFD+15 (13 cRA) in comparison to STD-C (*P*<0.01), STD+7.5 (13 cRA) (*P*<0.01) and STD+15 (13 cRA) (*P*<0.001) (Fig 8A).

An increase in kidney GSH levels was observed in HFD+7.5 (13 cRA) group compared to STD+7.5 (13 cRA) (*P*<0.001) and in HFD+15 (13 cRA) (*P*<0.05) compared to STD+7.5 (13 cRA) (*P*<0.05) and STD+15 (13 cRA) (*P*<0.05). A decrease in kidney GSH levels was observed in STD+15 (13 cRA) in relation to HFD+7.5 (13 cRA) (*P*<0.001) (Fig 8B).

An increase in kidney CAT activity was seen in HFD groups, with an increase in HFD+7.5 (13 cRA) in comparison to STD+7.5 (13 cRA) (*P*<0.05) and in HFD+15 (13 cRA) in comparison to STD-C (*P*<0.05), STD+7.5 (13 cRA) (*P*<0.01) and STD+15 (13 cRA) (*P*<0.05). A decrease in kidney CAT activity was seen in STD+7.5 (13 cRA) in comparison to HFD-C (*P*<0.05) (Fig 8C).

**Fig 9:**
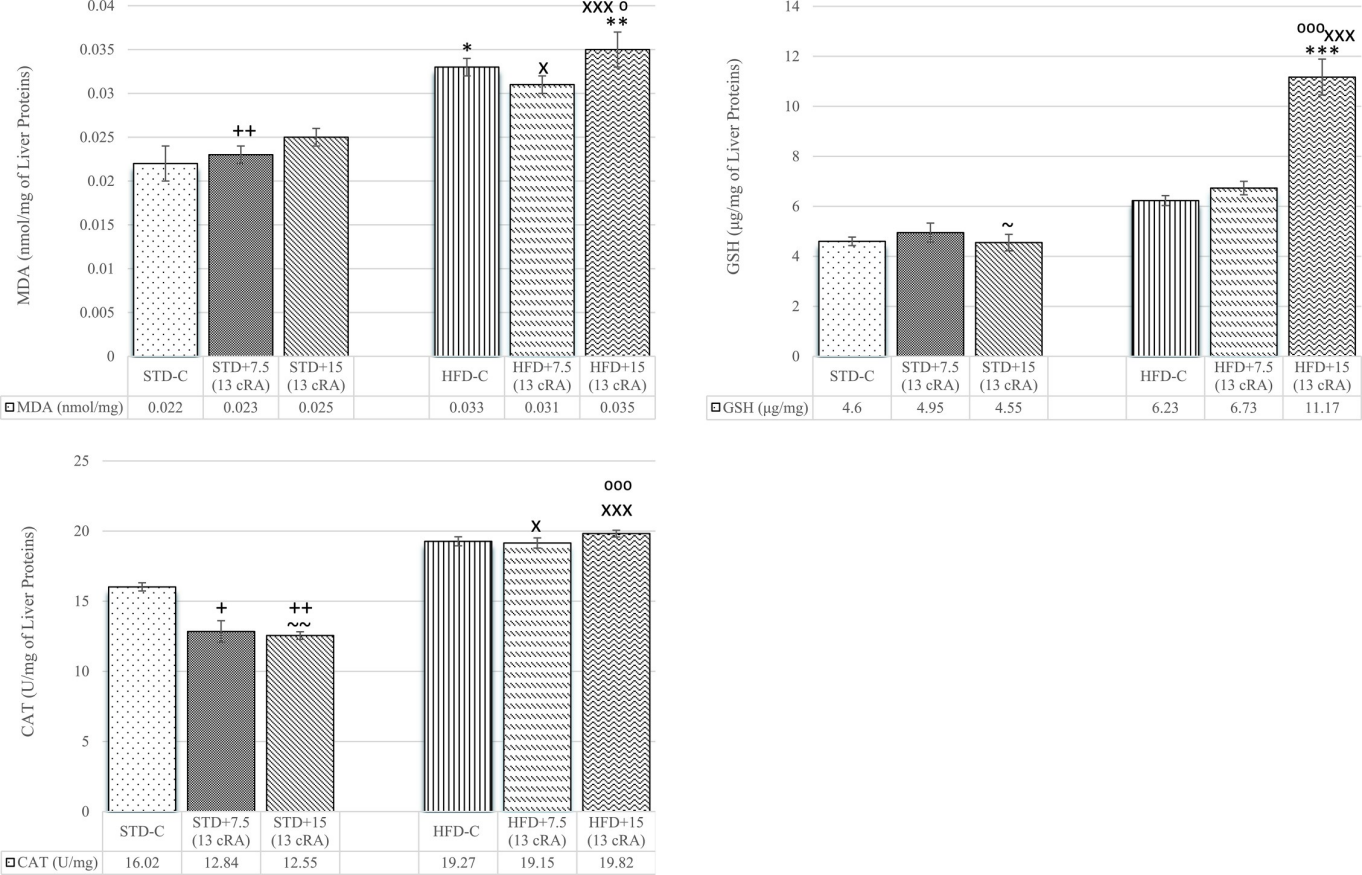
Effect of 13 cRA on MDA (A), GSH (B) and liver CAT activity (C) of rats fed standard or high fat diets. Rats were fed either STD or HFD for 9 weeks *ad libitum*. 13 cRA was orally administered to rats via a gastric tube in two different concentrations (7.5 mg/kg, 15 mg/kg) during 30 days. Liver was isolated when animals were sacrificed, on the 60^th^ day of the experiment. Number of rats per group: 6. *Different in relation to STD-C (* *P*<0.05; ** *P*<0.01; *** *P*<0.001). +Different in relation to HFD-C (+ *P*<0.05; ++ *P*<0.01). XDifferent in relation to STD+7.5 (13 cRA) (x *P*<0.05; xxx *P*<0.001). ^⁰^Different in relation to STD+15 (13 cRA) (^⁰^ *P*<0.05; ^⁰⁰⁰^ *P*<0.001). ~Different in relation to HFD+7.5 (13 cRA) (~ *P*<0.05; ~~ *P*<0.01). Data are expressed as mean ± SEM. Data were analysed using Kruskal-Wallis ANOVA (STATISTICA 13 StatSoft, Tulsa, OK, USA).

An increase in liver MDA levels was observed in groups on HFD, both HFD-C (*P*<0.05), HFD+7.5 (13 cRA) (*P*<0.05) and HFD+15 RA (*P*<0.05; *P*<0.01; *P*<0.001), respectively (Fig 9A).

An increase in liver GSH levels was seen in HFD+15 (13 cRA) compared to STD-C (*P*<0.001), STD+7.5 (13 cRA) (*P*<0.001) and STD+15 (13 cRA) (*P*<0.001) (Fig 9B).

An increase was seen in liver CAT activity of groups fed HFD with added 13 cRA: HFD+7.5 (13 cRA) compared to STD+7.5 (13 cRA) (*P*<0.05) and HFD+15 (13 cRA) compared to STD+7.5 (13 cRA) (*P*<0.001) and STD+15 (13 cRA) (*P*<0.001). A decrease in CAT activity was seen among groups fed STD depending on the dose of 13 cRA applied (*P*<0.05, *P*<0.01) (Fig 9C).

## Discussion

Obesity is one of the largest global health problems of the twenty-first century. We used a diet-induced animal model of obesity, which, according to a paper by Wong et al. [33], was developed to illustrate obesity in humans. This model imitates the effect of human obesity better than the genetic obesity model and is based on a diet with a high fat content leading to obesity, hyperglycaemia, and hyperlipidaemia. Vitamin A and its metabolites are important fat-soluble micronutrients for many essential physiological processes in a number of tissues and are also key regulators of the development of adipose tissue and associated metabolic complications. Many studies confirm the regulatory role of vitamin A and its metabolites in controlling obesity, but most of the vitamin A-mediated changes observed in human and animal models were contradictory [20]. Since there is no data on the consequences of long-term use of 13 cRA in early adolescence, present study was designed to identify the impact of 13 cRA supplementation, in combination with either STD or HFD, on body weight changes, adipose tissue distribution, glucose secretion, haematological and biochemical parameters, hormone disbalance, oxidative stress markers and atherogenic indices on Lewis rat model. When choosing rats, their age was taken into account so that it would correspond to human adolescence since young people are prone to eating high-calorie food and are exposed to the “obesogenic environment” on a daily basis, especially in western countries.

The results obtained showed that HFD diet with 13 cRA increased weight gain, visceral, retroperitoneal and perigonadal fat pad mass, adiposity index and leptin plasma levels but, on the other hand, decreased oral glucose tolerance compared to groups fed STD. HFD influenced the pattern of food and drink intake of animals (Table 2). Reduction in HFD consumption in relation to the STD diet in rats may be related to food composition and energy content, in which 45% of the energy comes from fat, which is in line with the findings of Marques et al. [34]. An increase in body weight percentage was detected in animals fed HFD with a higher dose of added 13 cRA, in comparison to STD or HFD control groups. A difference is seen from the fourth week till the end of the experiment. HFD control group of animals did not show difference in body weight change in relation to STD control group. In contrast, HFD animals administered 13 cRA in higher dose showed a significant increase in body weight, total body fat and adiposity index (Fig 1A and 1B; Table 2). That increase may be connected with the synergistic effect of 13 cRA and HFD. However, the aforementioned 13 cRA did not change the weight of the animals fed STD.

Weight gain is caused by an imbalance between energy uptake and energy expenditure that causes TG accumulation in white adipose tissue (WAT) [35] and other non-adipose tissues, such as muscle, liver, kidneys and heart [36]. Our data are consistent with that of Buettner et al. [37] and Ali Faran [38], who have shown that an increase in body weight can be observed after only four weeks of HFD, while other authors indicate that a longer period of high-fat diet intake is needed for weight changes in animals [39]. Crawford et al. showed that the HFD regime applied to 6-week old Spraque Dawley rats increased their body mass index (BMI) and fasting glucose levels [24], which was also confirmed in Wistar rats [40]. However, according to Woods et al., an increase in the percentage of adipose tissue in obese strains does not have to be accompanied by a significant weight gain [39].

Some research shows that retinoids can reduce body weight, subcutaneous and visceral fat of mice fed HFD [41]. A possible mechanism by which vitamin A reduces fat and body mass, as well as increases energy expenditure, is through enhanced thermogenesis and activation of uncoupling protein 1 (UCP1) located in the inner mitochondrial membrane of adipocytes. Moreover, the decreased thermogenic capacity of brown adipose tissue (BAT) is reported in some genetically obese animals [42]. In the study of Jeyakumar et al. vitamin A supplementation (129 mg/kg) resulted in the BMI decrease of obese rats, but without difference [18], which is opposed to our results. Our results are not consistent with already published studies which show that high levels of vitamin A supplementation lead to dose-dependent significant weight loss and decreased visceral adipose tissue [43]. However, our research is in line with another research by Jeyakumar et al. who demonstrated that the chronic supplementation of a vitamin A-enriched diet (retinyl palmitate) induces weight gain, adiposity, retroperitoneal adipose tissue and hypertriglyceridemia in both lean and obese rats of the WNIN/GR-Ob strain [44]. It is also in line with a research by Safonova et al. [19] who demonstrated that RA at low doses may promote adipogenesis. The weight gain of HFD-fed rats can be explained as side effects of retinoids, which cause the accumulation of WAT and consequently lead to an increase in body mass. According to Zhang et al. [6], HFD induced hyperlipidaemia affected activity and the expression of hepatic enzymes, such as alcohol dehydrogenases (ADHs), retinol dehydrogenases (RDHs) and retinal dehydrogenases (RALDHs), which reduced plasma retinol levels. On the other hand, it significantly increased retinol levels in tissues (liver, kidney, and adipose tissue) in rats, leading to obesity by decreasing plasma retinol levels in HFD rats. This is consistent with observations in obese humans. Thus, it seems that the regulation of adiposity/obesity by vitamin A and its metabolites depends on species, sex, age, duration and dosage, as suggested by Jeyakumar et al. [44].

In our study, it is possible that a longer period of a high fat diet would result in a greater difference in weight gain between individuals fed standard and high fat diet, as well as between groups with added 13 cRA. Nevertheless, the percentage of weight change over a 9-week period in female rats may be less pronounced due to the protective effect of oestrogen hormones, but also other hormones such as prolactin, thyroid hormone, and glucocorticoids, which can influence adipocytes in adipose tissue [45–47].

Weight gain is accompanied by an increase in TG and TC levels in experimental rats fed HFD, with or without added 13 cRA (Table 3). Specifically, WAT stores energy in the form of triglycerides as a single large lipid droplet. Excessive accumulation of triglycerides in tissues and organs causes morphological and anatomical changes and injuries such as pancreatic dysfunction (especially β-cells) and liver tissue, consequently leading to insulin resistance and non alcoholic fatty liver disease (NAFLD) [48]. Thus, obesity is associated with metabolic abnormalities connected to an increase in circulating levels of insulin and impaired insulin action, as well as to the dysregulation of adipokine secretion (an increase in leptin and decrease in adiponectin levels) (Figs 2 and 3). Elevated leptin levels are associated with insulin resistance (Fig 4E), hypertension, and cardiac hypertrophy. Thus, hypercholesterolemia and dyslipidaemia in the HFD group, together with 13 cRA supplementation, are closely associated with the pathogenesis of atherosclerosis. It has been reported that 13 cRA induces blood chemistry abnormalities in humans, such as elevated levels of TGs, TC, apolipoprotein B, calcium and creatine kinase activity, as well as hyperglycaemia [49, 50]. Some of these effects are apparent within a few days of treatment onset. Most of the biochemical changes in our study are consistent with the mentioned data, especially the increase in ALT, amylase activity, Ca and creatinine, as well as the decrease in ALB and total protein levels in the group fed HFD diet with 13 cRA added at a higher dose (Table 5). According to He et al., decreased Ca levels can cause suppression of certain calcium sensing enzymes required for lipolysis [51], which can lead to WAT accumulation. On the other hand, Parra et al. suggested that calcium intake decreases body weight and body fat gain in mice fed high-fat diet [52]. Increased serum amylase activity, other than pancreatitis, could be associated with increased absorption of carbohydrates, leading to enhanced energy intake and consequently, obesity. In STD fed rats, serum amylase concentration increases depending on the concentration of the administered 13 cRA, with no difference observed.

In the present study, the levels of TC, TG, LDL-c and VLDL-c were higher (Table 3) in the HFD groups compared to the STD groups, similarly to a previous study [4] where authors showed that elevated TC and TG levels may be a crucial factor in lipoprotein metabolism. Higher concentrations of TC and TG contribute to increased LDL-c formation and deposition, which is potently atherogenic. These data are consistent with our data on the increase in atherogenic indices (AIP, AC, CRR), and insulin resistance (Fig 4). It is well known that dyslipidaemia, LDL oxidation and low circulating levels of HDL are risk factors for coronary heart diseases [53].

Insulin resistance is an initiating disorder of type 2 diabetes. Nowadays, it is known that insulin resistance is not solely a consequence of glucose overflow in the blood due to its inability to enter a cell, but also of other numerous factors including hormones, adipokines and inflammatory cytokines [54]. An increase in circulating free fatty acids (FFA) is believed to play a pivotal role in the pathogenesis of insulin resistance, a common disorder in the metabolic syndrome [55]. As expected, 13 cRA and HFD impaired not only lipid metabolism, but also glucose metabolism, as confirmed by the OGTT test (Fig 5B). High-fat diet in combination with 13 cRA in two different concentrations (7.5 mg/kg and 15 mg/kg) shows an increase in glucose levels at the end of the test, in comparison to STD-C (*P*<0.01). The most likely reason for higher glucose concentrations is insulin resistance, but that cannot be confirmed in this study due to the fact that the measurement of insulin and insulin tolerance test were not performed. In this study, we used the TG/HDL-c ratio as the surrogate indicator for insulin resistance, as suggested in recent literature [56]. According to the literature, TG/HDL-C ratio may be a marker of cardio-metabolic risk and cardiovascular disease, as well as a useful parameter in hypertensive subjects [57]. Thus, TG/HDL-C ratio might be a useful predictor of glycaemic control in normal weight, as well as in overweight and obese patients and animals.

The connection between decreased glucose uptake and reduced insulin secretion, as well as insulin resistance, is confirmed in already published studies [58]. When developing insulin resistance, a decreased number of insulin receptors on cells are observed [58], as well as the number of glucose receptors, GLUT2, on pancreatic β cells [59]. Impaired intracellular signalling prevents glucose uptake into the cell [60]. 13 cRA administration to animals fed STD does not show alterations in glucose levels neither during the test, norat the test end (Fig 5A).

In addition, excessive energy supply leads to increased oxidative activity, as seen in liver and kidneys (Figs 8 and 9). Increased oxidative stress is accompanied by elevated leptin levels and TNF-α secretion, which promotes chronic inflammation. Thus, the raised leukocyte count may reflect a low-grade inflammation (Table 4), which was confirmed by the moderate neutrophilic leukocytosis in HFD fed rats with 13 cRA supplementation. According to Peelman et al., the mechanism responsible for leukocytosis in obesity, diabetes [61] and atherosclerosis may be leptin and leptin receptor, which exhibits structural similarity to class I cytokine receptors [62]. Interestingly, the gp130 subunit of the IL-6 receptor family also belongs to this class of receptors, suggesting that IL-6 and leptin may operate in a similar action mode [63], which may explain the raised leukocyte count. Polymorpho- and mononuclear leukocytes can be activated by increased glucose levels [64], oxidative stress [65] and cytokines [66], as well as other factors which can participate in the pathogenesis of micro- and macrovascular complications.

It is known that hyperleptinaemia and leptin resistance may upregulate the generation of ROS, increasing oxidative stress and promoting inflammation. Markers of kidney and liver oxidative stress are shown in Figs 8 and 9. Already published literature confirms that a high-fat diet leads to an increase in rat liver and kidney oxidative stress markers [67, 68], which is in accordance with our results. MDA is a stable product of lipid peroxidation, a cascade reaction of free radicals with lipids that lead to cell membrane destabilization. Increased MDA in liver follows an increase in CAT activity (Fig 9C) and GSH levels (Fig 9B), with difference in HFD+15 RA (*P*<0.05, *P*<0.01, *P*<0.001).

GSH levels have not increased in HFD-C and HFD+7.5 RA (Fig 9B), which is in accordance with other studies and which shows that increased oxidative stress does not have to be followed by increased GSH activity in liver and kidney cells [68].

However, there are many studies that emphasize 13 cRA effectiveness in lowering liver MDA production and generation, as well as oxygen consumption associated with lipid peroxidation in rat liver microsomes at concentrations as low as 25 mM [69]. Our results are not consistent with the above mentioned. In our study, 13 cRA does not exhibit a protective effect in oxidative stress development. On the contrary, 13 cRA in combination with HFD has a prooxidative effect, which is in accordance with some other studies [70].

It is possible that an increase in GSH in HFD-fed rats together with a high dose of 13 cRA in our data partially reduce the effects of oxidative stress, as indicated by a slight increase in adiponectin levels (Fig 3), which is in line with the study by Heliovaara et al. [71]. However, this insignificant increase is not sufficient to reduce insulin resistance and atherosclerotic and cardiovascular diseases (Figs 4A–4C, 5A and 5B).

Considering all the above mentioned, we can conclude that HFD diet with 13 cRA synergistically increased weight gain, total body fat, adiposity index, dyslipidaemia, hyperleptinaemia, insulin resistance, VLDL-c concentrations, oxidative stress and atherogenic indices, as well as decreased oral glucose tolerance compared to groups fed STD. However, 13 cRA in combination with STD did not induce remarkable metabolic changes, only slightly increased TG, TC and atherogenic parameters. According to our data, it seems that the impact of 13 cRA with regards to obesity and metabolism is quite complex. Many other factors, such as dose, genetics, age, sex, adipose tissue distribution and kidney function have to be taken into consideration. It appears that the administration of 13 cRA may have a completely different effect on metabolism in rats fed STD or HFD, so further research should focus on immune and histopathological alterations within tissues to further confirm the negative effect of 13 cRA in HFD-fed Lewis rats. Given the data obtained on the use of 13 cRA in animals, it seems that the use of Isotretinoin in the treatment of acne should be taken with caution given the possible consequences during the later stages of life.
